# Fruitful or unfruitful: strawberry and tomato specific immunoglobulin E testing at a tertiary pediatric center

**DOI:** 10.3389/falgy.2023.1277631

**Published:** 2023-10-16

**Authors:** Albert C. Chong, Neema Izadi, Won Jong Chwa, Jonathan S. Tam

**Affiliations:** ^1^ Keck School of Medicine, University of Southern California, Los Angeles, CA, United States; ^2^ Division of Clinical Immunology and Allergy, Children’s Hospital Los Angeles, Los Angeles, CA, United States; ^3^ Saint Louis University School of Medicine, Saint Louis University, Saint Louis, MO, United States

**Keywords:** food allergy, strawberry, tomato, immunoglobulin E, pediatric

## Abstract

**Background:**

Suspected strawberry and tomato (S/T) food allergy (FA) can be evaluated using specific immunoglobulin E (sIgE) testing despite its low specificity and positive predictive value.

**Objective:**

This study aims to understand ordering patterns for S/T sIgE testing and identify relevant factors to clinical decision-making.

**Methods:**

We retrospectively reviewed 814 patients with sIgE testing available for strawberries (651), tomatoes (276), or both (113) from January 2012 to May 2022 at a tertiary pediatric hospital. Patient demographics, provider specialty, and reasons for testing were collected. Student’s *t*-test and multiple regression analyses were performed to test the association between the S/T sIgE level and clinically relevant outcome (CRO) status. Fisher’s exact test and general linear models were used to evaluate and compare potential predictive factors for CRO status.

**Results:**

Allergy and immunology, gastroenterology, and general pediatrics ordered most S/T sIgE testing. Testing was ordered most frequently for non-IgE-mediated gastrointestinal symptoms, mild possible IgE-mediated reactions, and eczema. Testing was most often ordered for infants and school-age children. Mean sIgE levels were higher for S/T tests resulting in a CRO when controlling for other predictor variables (*p* = 0.015; *p* = 0.002 for S/T, respectively). Only 2.2% and 5.4% of tests resulted in a CRO for S/T, and severe allergy was rare. Testing for non-IgE-mediated GI symptoms or eczema, or in non-atopic patients, yielded no CROs. Exposure and reaction history of present illness (ERH) was associated with CROs (*p* < 0.001; *p* = 0.04) with a high negative predictive value (99.5%; 100%) and low positive predictive value (11.5%; 15.0%). ERH (*p* < 0.001, *η*^2^ = 0.073; *p* = 0.009, *η*^2^ = 0.123) was a more significant predictor than the sIgE level (*p* = 0.002, *η*^2^ = 0.037; *p* = 0.212, *η*^2^ = 0.030) for CRO status.

**Conclusion:**

The diagnosis of S/T food allergy is made primarily based on clinical history. S/T sIgE testing for children and adolescents should be avoided for patients without an ERH and in the workup of non-IgE-mediated GI symptoms. Testing for eczema and non-atopic patients is likely low-yield.

## Background

According to self-reported measures, food allergy (FA) is a major public health concern, affecting one in 13 children and one in 10 adults in the United States (US) (1, 2). Strawberry and tomato (S/T) are frequent suspects for FA as they are commonly consumed worldwide; however, these fruits may cause many non-allergic or local irritant reactions because they are highly acidic and may even cause pseudoallergic histamine release (3, 4). While accurate prevalence data are essential for developing effective strategies to prevent and manage FA, obtaining these measures is a complex and challenging task. The prevalence of strawberry allergy has been reported as 0.5%–4% in childhood (5); meanwhile, tomato allergy may account for 1.5% of FA in Northern Europe (6, 7). Severe reactions to S/T have rarely been described (8–10), and S/T allergy presents more often as pollen FA syndrome (PFAS), which is usually mild (11). A major mechanism of S/T PFAS is thought to involve cross-reactivity of strawberry Fra a 1 or tomato Sola l 4 to Bet v 1 in birch-pollen sensitized individuals, which may represent 8%–16% of Europe’s population and more than 100 million persons globally (12–15).

Specific immunoglobulin E (sIgE) testing is often ordered in the FA workup but may not always be appropriately applied given its low specificity and positive predictive value (16, 17). While identifying FA is critical for preventing life-threatening reactions, overdiagnosis may result in physical, mental, and financial consequences for patients and their caregivers (18–22). For example, food avoidance can lead to nutritional problems or the development of FAs from a delayed introduction of allergenic foods (23–25). Meanwhile, patients and caregivers may suffer a worse quality of life due to the daily challenge of acquiring allergen-free foods, fear of accidental exposure, and bullying of food-allergic children (22, 26, 27). Further, unnecessary testing increases medical costs. Per food, sIgE testing costs $15–$35 in the US and £15–£100 in the United Kingdom (28). The need for special foods and epinephrine autoinjectors greatly magnifies the financial burden of FAs (21, 29, 30).

Given the significant consequences of FA misdiagnosis, highly predictive sIgE thresholds would be greatly beneficial. Some thresholds have been determined for the most common food allergens (i.e., milk, egg, and peanut), and component testing has further improved diagnostic accuracy, particularly for peanut allergy (20, 31, 32). However, sIgE testing for S/T remains poorly studied. Recently, a retrospective study conducted in Spain studied 43 children with a self-reported history of strawberry allergy. Among these children, 67% had positive strawberry allergy testing, but 94% tolerated the fruit, bringing into question the relevance of reported reactions and testing (33). A similar study for tomato allergy has not been reported. To improve quality and value in FA care, we aim to understand provider ordering patterns for S/T sIgE testing and identify clinically relevant factors to guide decision-making for these tests.

## Methods

### Specific immunoglobulin E testing specifications

All sIgE testing at our institution was performed on a Phadia 250 instrument (Thermo Fisher, Article #12-3900-01) using three positive controls (pooled human samples containing sIgE antibodies to house dust mite, common silver birch, or cat dander, respectively, for the high, medium, and low controls, all in 0.05% sodium azide) and one negative control (pooled human samples in 0.05% sodium azide without antibodies). The lower bound of testing was <0.35 kU/L prior to 2020 and <0.10 kU/L thereafter per manufacturer update. The upper bound was 100 kU/L for all samples.

### Data collection

We retrospectively reviewed 814 patients who had sIgE testing performed for strawberry (651), tomato (276), or both (113) from January 2012 to May 2022 at Children’s Hospital Los Angeles (CHLA). For patients with multiple S/T sIgE tests, the most recent test was used for analysis. Age at testing was grouped into one of five categories: infant (0–2 years old), preschool (3–6 years old), school age (6–12 years old), adolescence (13–17 years old), and adult (18 years old and over). Reasons for sending to S/T sIgE testing were determined from the ordering encounter note when available or the electronic test order form. We identified one or more testing reasons for each case using 10 categories: eczema, mild possible IgE-mediated reaction (i.e., hives, non-specific rash, and eye itching), severe possible IgE-mediated reaction (i.e., anaphylaxis), non-IgE-mediated gastrointestinal symptoms (i.e., abdominal pain, constipation, and diarrhea), PFAS (i.e., lip swelling and throat itching), unspecified reaction to S/T, previous positive S/T testing without a history of reaction, history of other food allergies, parental request otherwise not indicated, and unknown.

### Data processing

**Figure 1:**
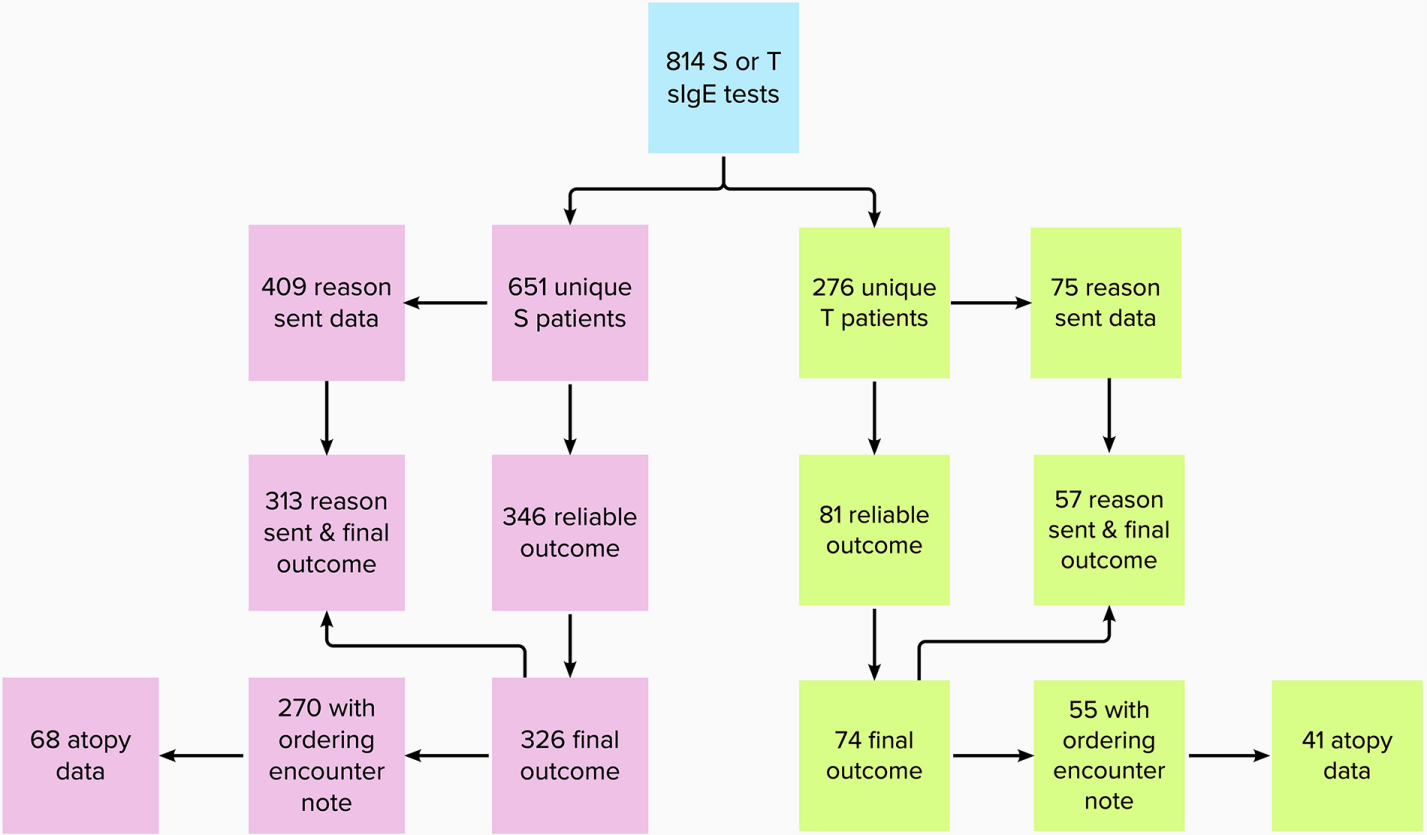
Flowchart of available data for each strawberry and tomato test.

Data were filtered systematically (Figure 1) for whether sIgE testing was for strawberry or tomato allergy, reliability of outcome data (i.e., a clear recommendation from any specialty regarding S/T following S/T sIgE testing), documentation of a final outcome (i.e., patient told to avoid the tested food or patient recommended/allowed to continue eating the food), availability of rationale, availability of the provider note from the ordering encounter, and availability of atopy data. For diagnostic yield analyses, we defined a clinically relevant outcome (CRO) as a definitive outcome where the patient was ultimately told to avoid S/T or consume cautiously due to PFAS. For analyses involving testing rationale, all reasons for sending a single test were considered to maximize fidelity; this was accomplished by treating each reason documented as a separate testing data point. To explore the relevance of an exposure and reaction history of present illness (ERH), patients were positive for ERH if the patient (themselves or via caregiver) reported an exposure to the specific food (ingestion or cutaneous contact) with a subsequent reaction during the ordering encounter (no specific time to reaction was required). Patients were considered atopic if they were diagnosed with eczema, asthma, and/or allergic rhinitis by an allergy and immunology (A/I) specialist at our hospital.

### Statistical methods

Statistical analyses were conducted using SPSS (version 28, 2022) and Prism (version 9, 2022). Student’s *t*-test was used to compare the mean sIgE levels between CRO and non-CRO groups for each food. (“<0.10” and “<0.35” were converted to “0.35” for quantitative analysis, given different minimum thresholds before and after 2020.) Multiple regression was then performed to compare mean sIgE levels between CRO and non-CRO groups while controlling for age at testing, sex, ethnicity, and ordering specialty. Fisher’s exact test was used to evaluate associations between potential predictive factors (i.e., ERH and atopy) and having a CRO following S/T sIgE testing. A general linear model was employed to calculate partial eta-squared effect sizes for comparing the effect of ERH vs. S/T sIgE testing on CROs.

## Results

### Demographics

**Figure 2:**
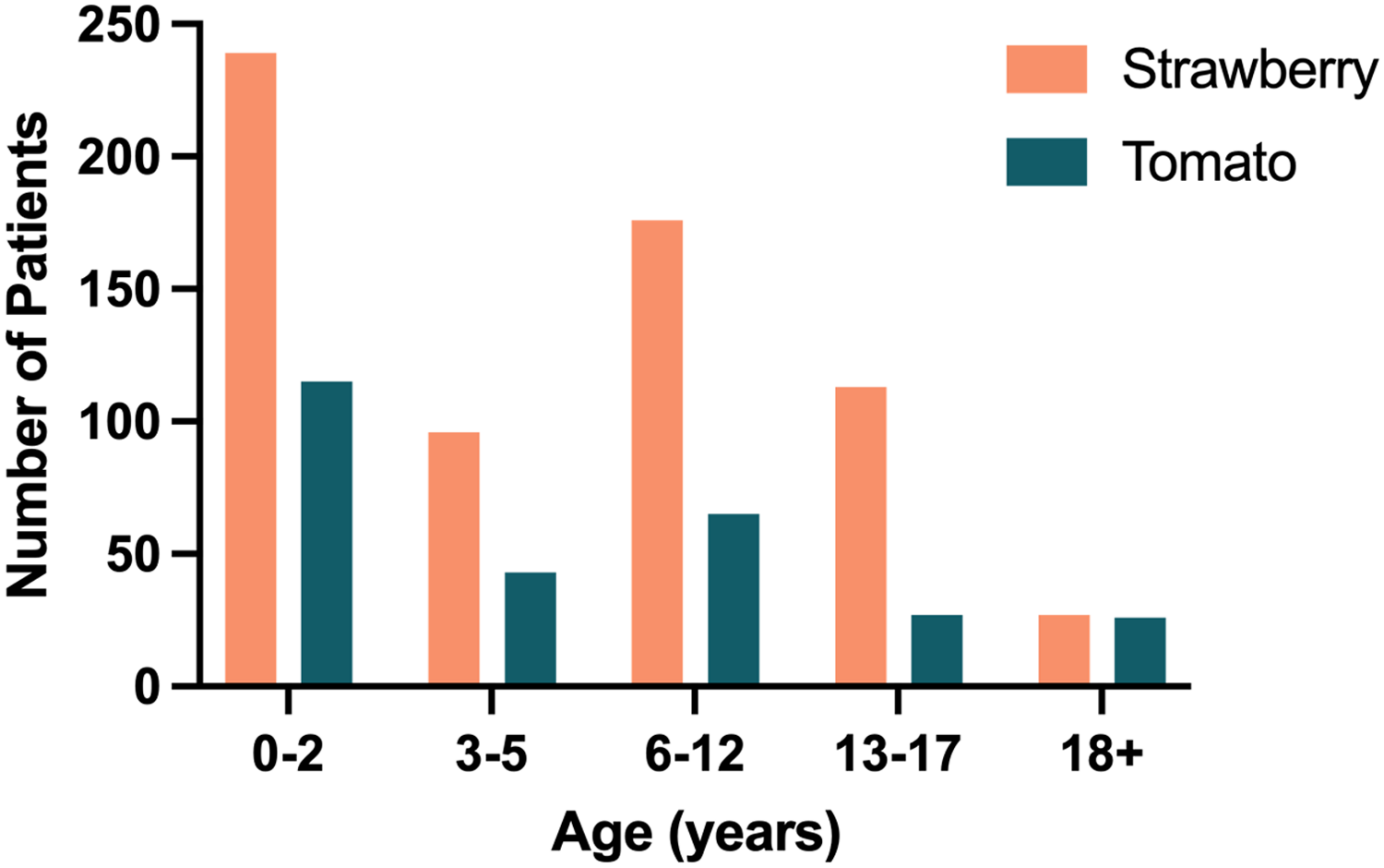
Number of patients in each age group with strawberry and tomato testing.

Our sample included 51.5% male participants for strawberry testing and 56.5% male participants for tomato testing. The mean age at testing was 7.7 ± 8.1 years for strawberry allergy and 8.8 ± 12.6 years for tomato allergy. Age distribution was bimodal, with the largest peak seen for infants (0–2 years old) (36.7%; 41.7%) and a second smaller peak observed for school-age children (6–12 years old) (27.0%; 23.6%) (Figure 2). Adults represented the smallest fraction for both S/T (4.1%; 9.4%) tests. Race/ethnicity for strawberry testing was 28.9% White, 4.3% Black, 7.1% Hispanic/Latino, 6.0% Asian/Pacific Islander, 1.2% multiple, 20.1% other, and 32.4% unknown; for tomato testing, race/ethnicity was 22.8% White, 5.4% Black, 7.2% Hispanic/Latino, 5.8% Asian/Pacific Islander, 0.4% multiple, 22.5% other, and 35.9% unknown. Inpatients represented only 0.8% (5/651) and 1.4% (4/271) of S/T sIgE tests, respectively. Atopy was present in 60.6% (86/142) of patients tested for strawberry allergy and 61.2% (52/85) of patients tested for tomato allergy seen and with testing ordered by A/I specialists at CHLA.

### Ordering provider specialties

**Figure 3:**
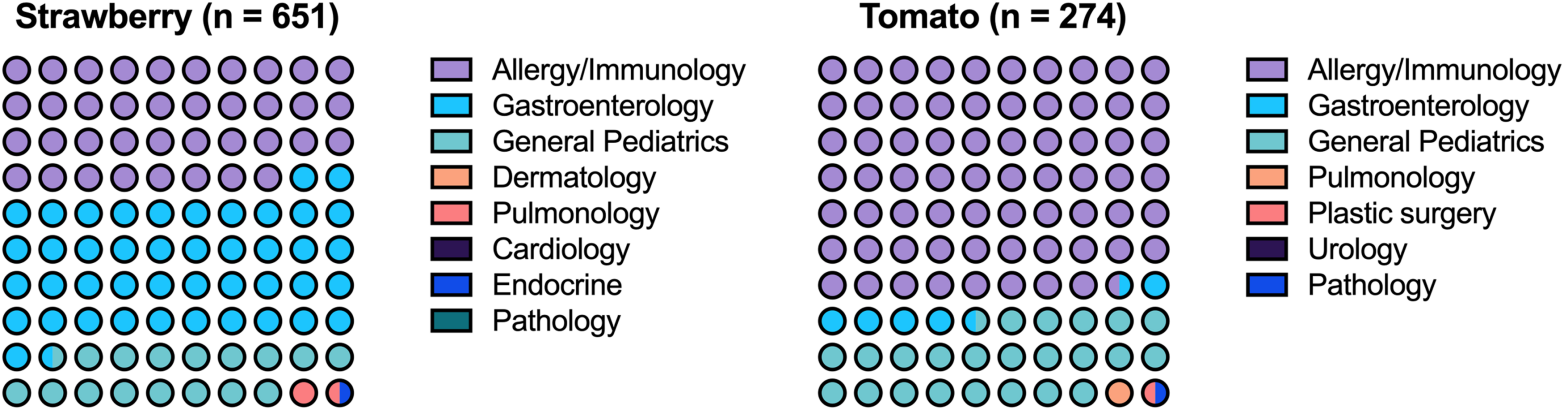
Specialty ordering strawberry and tomato testing.

The specialty of the ordering provider was available for 651 strawberry and 274 tomato tests (Figure 3). A/I, GI, and general pediatrics (GP) were the top ordering specialties, accounting for 97.8% and 98.2% of S/T sIgE tests, respectively. For strawberry allergy, GI (43.0%) ordered the most tests, followed by A/I (38.2%) and GP (16.6%). For tomato allergy, A/I (68.7%) ordered the most tests, followed by GP (23.7%) and GI (5.8%).

### Reasons for testing

**Figure 4:**
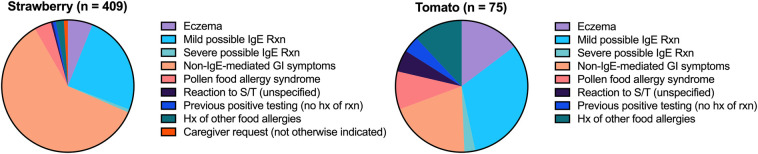
Reasons for strawberry and tomato testing.

Reasons for sIgE testing were available for 409 strawberry and 75 tomato tests (Figure 4). Non-IgE-mediated gastrointestinal symptoms, mild possible IgE-mediated reactions, and eczema were the top reasons, accounting for 90.9% of strawberry and 66.7% of tomato tests. For strawberry allergy, non-IgE-mediated gastrointestinal symptoms (60.1%) were the most common reason for testing, followed by mild possible IgE-mediated reactions (24.7%) and eczema (6.1%). For tomato allergy, mild possible IgE-mediated reactions (32%) were the most common reason, followed by non-IgE-mediated gastrointestinal symptoms (20%) and eczema (14.7%). PFAS symptoms (4.2%; 9.3%) and a history of other food allergies (2.0%; 12%) were the next most common reasons for S/T testing, respectively.

### CRO and epinephrine use

Final outcome data were available for 326 strawberry and 74 tomato cases. Of these, 2.15% (7/326) and 5.41% (4/74) of S/T sIgE tests resulted in a CRO. PFAS represented 42.9% (3/7) and 50% (2/4) of these CROs for S/T, respectively. For the four non-PFAS cases where the patient was ultimately told to avoid strawberries, reasons for avoidance included anaphylaxis, rash, unspecified acute reaction, and concern for strawberry allergy due to unspecified blueberry allergy. For the two non-PFAS tomato cases, rash (specifically eczema and hives) and unspecified acute reaction were the reasons for avoidance. Only one patient required epinephrine administration for strawberry allergy (0.3%, 1/326), and epinephrine was never required for tomato allergy (0/74).

### sIgE levels and CRO

sIgE levels were available for all cases with final outcome data (7 and 4 with CROs and 319 and 70 without CROs for S/T, respectively). The mean sIgE levels for S/T tests resulting in CROs were 5.5 ± 5.8 kU/L and 12.5 ± 1.7 kU/L, respectively. For tests without CROs, the mean sIgE levels were 0.9 ± 4.0 kU/L and 1.8 ± 3.7 kU/L, respectively. For both foods, the mean sIgE level was significantly higher among S/T tests resulting in CROs vs. no CROs (*p* = 0.003; *p* < 0.001 for S/T, respectively). Multiple linear regression models, including CRO status and other covariates, significantly predicted sIgE levels for strawberry [F(5,320) = 3.730, *p* = 0.003] and tomato [F(5,68) = 2.633, *p* = 0.031] allergy (Supplementary Material A). For strawberry allergy, CRO status (*p* = 0.015) and ordering specialty (*p* = 0.015) predicted sIgE levels, while for tomato allergy, only CRO status (*p* = 0.002) predicted sIgE levels.

### Age and CRO

Age at testing was available for all cases with final outcome data. Following strawberry sIgE testing, CROs were observed for all age groups prior to adulthood: 2.3% (2/88) for infants, 4.3% (2/47) for preschool children, 1.0% (1/99) for school-age children, 2.4% (2/83) for adolescents, and 0% (0/9) for adults. One infant and two preschool children accounted for the three strawberry PFAS outcomes. Following tomato testing, CROs were observed for 7.1% (2/28) of school-age children and 18.2% (2/11) of adolescents but not for any other age group (0/17 for infants, 0/12 for preschool children, and 0/6 for adults). Adolescents accounted for the only two PFAS outcomes after tomato sIgE testing.

### Reason to test and CRO yield

Filtering for cases with a documented reason to test and final outcome data resulted in 313 strawberry and 57 tomato cases (Table 1). S/T sIgE testing for non-IgE-mediated GI symptoms or eczema resulted in no CROs. Testing for PFAS symptoms yielded 21.4% and 40% CROs for S/T, respectively. Testing for mild and severe possible IgE-mediated reactions resulted in some CROs for strawberry allergy but none for tomato allergy.

**Table 1:** CRO yield by reason for strawberry and tomato testing.

Reason for testing	Strawberry (*n* = 313)			Tomato (*n* = 57)		
	Number of patients	Number with CRO	CRO yield (%)	Number of patients	Number with CRO	CRO yield (%)
Eczema	17	0	0	10	0	0
Mild possible IgE reaction	78	3	4	16	0	0
Severe possible IgE reaction	3	1	33	2	0	0
Non-IgE-mediated gastrointestinal symptoms	188	0	0	12	0	0
Pollen food allergy syndrome	14	3	21	5	2	40
Reaction to S/T (unspecified)	1	1	100	3	1	33
Previous positive testing (no history of reaction)	3	0	0	2	0	0
History of other food allergies	5	1	20	7	0	0
Caregiver request (not otherwise indicated)	4	0	0	0	0	n/a

### Exposure and reaction history and CRO

**Figure 5:**
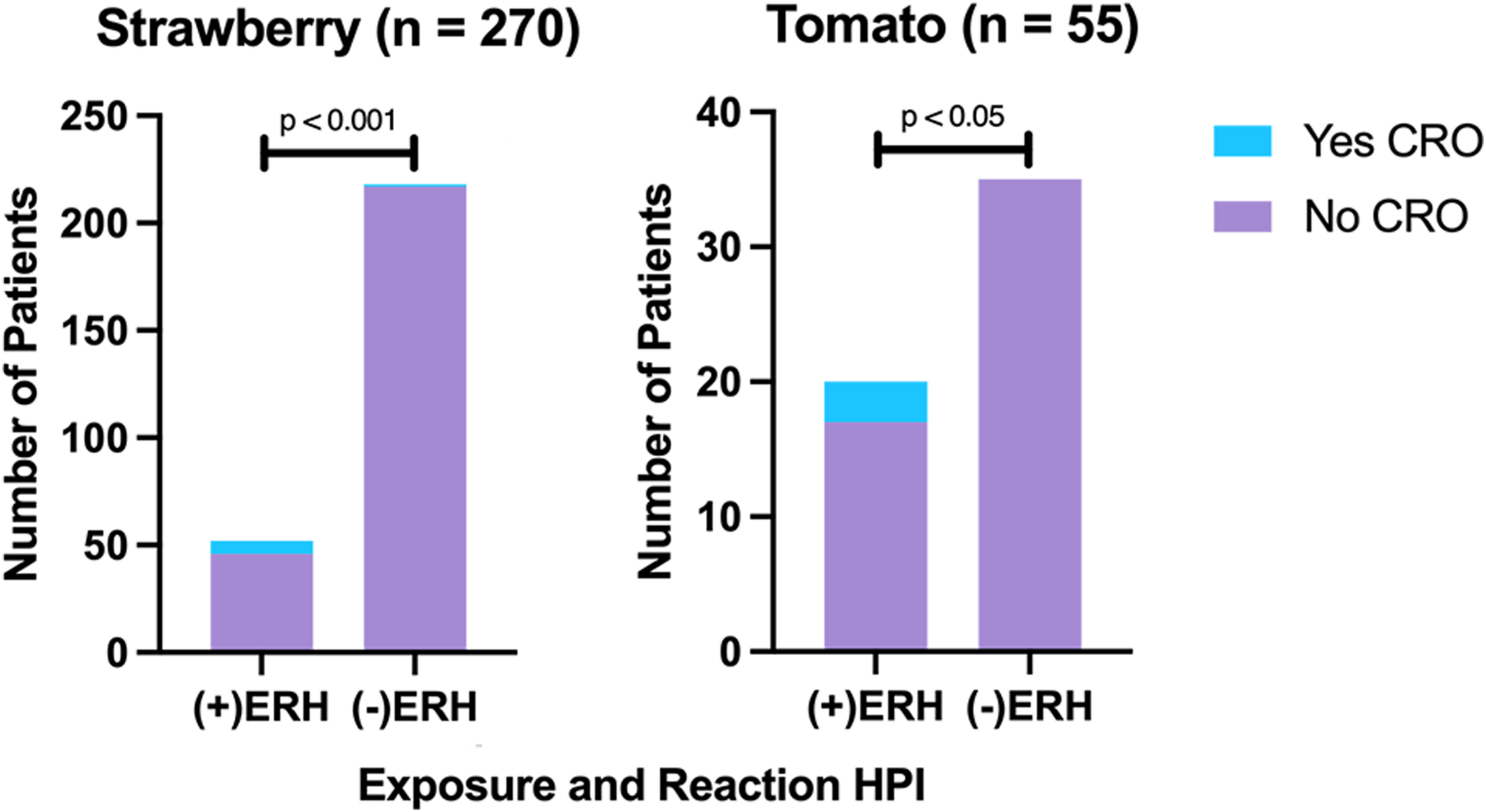
ERH was highly associated with CROs for strawberry and tomato testing.

An ordering encounter note and final outcome data were available for 270 strawberry and 55 tomato tests. ERH was significantly associated with a CRO following sIgE testing for S/T (*p* < 0.001; *p* = 0.04) with a high negative predictive value (99.5%; 100%) and a low positive predictive value (11.5%; 15.0%) (Figure 5). For strawberry cases, ERH significantly predicted CROs (*p* < 0.001) with a medium effect size (*η*^2^ = 0.073), while sIgE levels also significantly predicted CROs (*p* = 0.002), albeit with a small effect size (*η*^2^ = 0.037). For tomato cases, ERH significantly predicted CROs (*p* = 0.009) with a large effect size (*η*^2^ = 0.123), while sIgE levels did not (*p* = 0.212, *η*^2^ = 0.030).

### Atopy and CRO

An ordering encounter note and final outcome data from A/I providers at our hospital were available for 68 strawberry and 41 tomato cases. Among this subgroup of cases, atopy was present in 75.0% (51/68) of strawberry and 78.0% (32/41) of tomato patients. None of the non-atopic S/T patients had a CRO following S/T sIgE testing (0/17; 0/9 for S/T, respectively). Meanwhile, 11.8% (6/51) and 9.4% (3/32) of atopic patients had a CRO following S/T testing, respectively. The association between atopic status and CRO following S/T sIgE testing was not statistically significant even when both were considered together (*p* = 0.11).

## Discussion

Our study characterized the ordering patterns for more than 800 S/T sIgE tests at a tertiary pediatric hospital over 10 years and assessed factors that may influence diagnostic yield. S/T sIgE testing was most frequent for infants, reflecting caregiver and provider prioritization of establishing allergenic triggers with initial food introduction. Testing decreased for preschool children before increasing again for school-age children, which may reflect the increased risk of exposure to new foods with the start of school. At our hospital, S/T sIgE testing was almost exclusively used by A/I, GI, and general pediatrics and rarely by any other specialty. For both foods, mild possible IgE-mediated reactions, non-IgE-mediated gastrointestinal symptoms, and eczema were the most frequent reasons for testing.

Mean sIgE levels were significantly higher for S/T tests with a CRO, yet diagnostic yield was quite low, with only 2.2% (7/326) and 5.4% (4/74) of tests yielding a CRO. While the prevalence of S/T allergy was low in our sample, it is likely even lower in the general population. Patients in this study were suspected to have S/T allergy before testing and thus had an elevated pretest probability for a CRO. Further, atopy is associated with food allergy (34–37), and the presence of atopy in our sample was 2–8-fold higher than in the general population of developed countries (10%–30%) (38, 39). Perhaps expectedly, PFAS represented more than 40% of these CROs following S/T sIgE testing for both foods. Given the low CRO yields and rare S/T reactions in our cohort, more judicious testing for these foods should be considered.

Strikingly, non-IgE-mediated gastrointestinal symptoms accounted for more than 60% of strawberry tests despite no current evidence suggesting the benefit of sIgE testing for non-IgE-mediated gastrointestinal symptoms. Specialties other than A/I accounted for the majority of tests. Given that IgE testing for non-IgE-mediated gastrointestinal symptoms is contrary to known pathophysiology, it is unsurprising that none of the 188 strawberry sIgE tests sent for non-IgE-mediated gastrointestinal symptoms with final outcome data resulted in a CRO. This underscores the need for education on the situational value of IgE testing to specialties outside of A/I. Non-IgE-mediated gastrointestinal symptoms accounted for 20% of tomato tests. As with strawberry cases, this testing yielded no CROs. These findings highlight an opportunity to optimize ordering patterns by avoiding S/T sIgE testing for GI symptoms that are unlikely to be IgE-driven.

Atopic dermatitis (AD) is another condition for which S/T sIgE testing may not be helpful. Recent evidence suggests a limited role for sIgE testing (40) in AD management and mixed results from anti-IgE therapies (41). Of 17 strawberry and 10 tomato sIgE tests sent for “atopic dermatitis” or “eczema,” none resulted in a CRO. These results support the current understanding that food is not the primary cause of AD (42) and that dietary elimination for AD has little benefit and more potential for harm (40). Meanwhile, patients with AD tend to have higher sIgE levels to many allergens, many of which may have no clinical relevance, which reduces any utility of sIgE testing in AD (43). In this context, our data support that S/T sIgE testing is not indicated in the evaluation of most eczema.

S/T testing for PFAS symptoms and strawberry testing for possible IgE-mediated reactions had some CROs. CROs were expected when testing for PFAS symptoms for S/T, given that fruits are common triggers of PFAS, which is IgE-mediated (44, 45). However, PFAS may be diagnosed from clinical history and testing for pollen sensitization and generally does not require testing for specific foods. Meanwhile, testing for mild and severe possible IgE-mediated reactions resulted in only a small number of CROs for strawberry allergy and none for tomato allergy, suggesting that S/T infrequently cause IgE-mediated allergy beyond PFAS.

From an age standpoint, tomato sIgE testing resulted in no CROs for children under 11 years old (0/29), suggesting that tomato sIgE testing may not be of value for infants and preschool children. Furthermore, tomato sIgE resulted in PFAS only with the start of adolescence, consistent with previous observations that PFAS is less common in younger children (45). These trends were not observed for strawberry allergy, for which CROs were found for all age groups prior to adulthood and PFAS was found in patients under 6 years old and even in infants.

We also evaluated the potential relevance of an ERH or atopy in the decision to order S/T testing. Interestingly, the absence of an ERH made a CRO following S/T sIgE testing statistically unlikely, with high negative predictive values for both strawberry (99.5%) and tomato (100%) fruits. Alternatively, the presence of an ERH could not reliably predict a CRO with low positive predictive values for both strawberry (11.5%) and tomato (15.0%) fruits. Thus, a negative ERH may largely rule out S/T allergy. Furthermore, results showed that ERH rather than sIgE testing had a greater bearing on CRO status, revealing the importance of taking a good clinical history and that, in many cases, sIgE testing may not affect management. Regarding atopy, non-atopic patients were never found to have a CRO following testing (0/26). While this is consistent with increased atopy with FA, our study was underpowered to evaluate this association due to a lack of relevant data.

### Limitations

Our report had several limitations. Owing to the retrospective nature of this study and our relatively strict systematic approach to analysis, data were not always available. Racial/ethnic composition was also unclear in many instances. Regarding the reasons for testing analysis, since multiple reasons could be provided for a single sIgE test, we could not ascertain which of these reasons was linked to CROs in these cases. Finally, since our sample was largely pediatric, extrapolation of these findings to the adult population is limited.

## Conclusion

At a large tertiary pediatric center, S/T sIgE testing was predominately ordered by A/I, GI, and GP, mostly for infants and school-age children. Clinical history rather than serological testing was the primary determinant of CRO status. ERH had a very high negative predictive value but a poor positive predictive value for CROs. Although mean sIgE levels were higher for S/T tests resulting in CROs, the overall yield was quite low. A large number of strawberry tests were sent for non-IgE-mediated gastrointestinal symptoms with no CROs. Further, S/T tests may have minimal yield for patients without atopy and in the workup of most eczema, although larger studies are needed to confirm these additional findings. Taken together, the threshold for ordering sIgE testing for S/T may need to be re-evaluated.

## Data Availability

The original contributions presented in the study are included in the article/Supplementary Material, further inquiries can be directed to the corresponding author.

## Supplementary material

Supplementary Table S1 Specific immunoglobulin E level regression models.
