# Supplementary material for: Fruitful or unfruitful: strawberry and tomato specific immunoglobulin E testing at a tertiary pediatric center
Source: Front Allergy. 2023 Oct 16;4:1277631. doi: 10.3389/falgy.2023.1277631 (PMC10613731; doi:10.3389/falgy.2023.1277631)
Supplement: Supplementary Table S1 — Specific immunoglobulin E level regression models. [file Table1.docx]

| **A. Strawberry Specific Immunoglobulin E Level Regression Model** | | | | | |
| --- | --- | --- | --- | --- | --- |
| **ANOVA** | | | | | |
| Model | Sum of squares | df | Mean square | F | Sig. |
| Regression | 292.782 | 5 | 58.556 | 3.730 | 0.003 |
| Residual | 5024.134 | 320 | 15.700 |  |  |
| Total | 5316.916 | 325 |  |  |  |
| **Coefficients** | | | | | |
| Model | B | Standard error | Beta | t | Sig. |
| (Constant) | 1.219 | 0.522 |  | 2.337 | 0.020 |
| Ethnicity | 0.077 | 0.087 | 0.048 | 0.878 | 0.380 |
| Sex | 0.478 | 0.446 | 0.059 | 1.072 | 0.285 |
| Age at Strawberry Testing | -0.021 | 0.038 | -0.032 | -0.570 | 0.569 |
| Ordering Specialty | -0.898 | 0.367 | -0.135 | -2.445 | 0.015 |
| Clinically Relevant Outcome | 3.770 | 1.537 | 0.135 | 2.454 | 0.015 |
|  |  |  |  |  |  |
| **B. Tomato Specific Immunoglobulin E Level Regression Model** | | | | | |
| **ANOVA** | | | | | |
| Model | Sum of squares | df | Mean square | F | Sig. |
| Regression | 218.780 | 5 | 43.756 | 2.633 | 0.031 |
| Residual | 1130.079 | 68 | 16.619 |  |  |
| Total | 1348.859 | 73 |  |  |  |
| **Coefficients** | | | | | |
| Model | B | Standard error | Beta | t | Sig. |
| (Constant) | 2.138 | 1.028 |  | 2.080 | 0.041 |
| Ethnicity | -0.010 | 0.199 | -0.005 | -0.048 | 0.962 |
| Sex | -0.056 | 0.973 | -0.007 | -0.058 | 0.954 |
| Age at Tomato Testing | -0.066 | 0.059 | -0.130 | -1.130 | 0.263 |
| Ordering Specialty | -0.496 | 0.540 | -0.105 | -0.919 | 0.361 |
| Clinically Relevant Outcome | 6.973 | 2.142 | 0.369 | 3.255 | 0.002 |

**Supplemental A.** Multiple regression of predictors for specific immunoglobulin E levels in (A) strawberry and (B) tomato food allergy. ANOVA = analysis of variance. B = unstandardized Beta coefficient. df = degrees of freedom. Sig. = significance.
